# Metasurface‐Enabled Helical Wavefront Fingerprint Metrology for Quantitative Retrieval of Liquid Optical Constants

**DOI:** 10.1002/advs.77099

**Published:** 2026-08-24

**Authors:** Yuting Zhou, Hongliang Li, Simeng Li, Feiyu Jiao, Yansong Du, Jin Tae Kim, Sang‐Shin Lee, Duk‐Yong Choi, Fei Ding, Xuechao Yu, Xun Guan

**Affiliations:** ^1^ Tsinghua Shenzhen International Graduate School Tsinghua University Shenzhen China; ^2^ Key Laboratory of Multifunctional Nanomaterials and Smart Systems Suzhou Institute of Nano‐Tech and Nano‐Bionics Chinese Academy of Sciences Suzhou China; ^3^ Quantum Technology Research Department Electronics and Telecommunications Research Institute Daejeon South Korea; ^4^ Department of Electronic Engineering Kwangwoon University Seoul South Korea; ^5^ Department of Quantum Science and Technology Research School of Physics Australian National University Canberra Australia; ^6^ School of Electronic Science and Technology Eastern Institute of Technology Ningbo China

**Keywords:** extinction coefficient, helical wavefront fingerprint, liquid‐state photonics, metasurface, optical metrology, refractive index

## Abstract

Optical constants of liquids encode intrinsic signatures of molecular composition and electromagnetic response, forming the physical basis for quantified optical analysis. However, conventional ellipsometric measurements are fundamentally constrained by interface‐induced beam distortion arising from gas‐liquid meniscus effects that limit the measurement precision and reliability, and necessitate bulky optical instrumentation. Here, we propose and experimentally validate a Metasurface‐enabled Helical Wavefront Fingerprint (MHWF) metrological paradigm for quantitatively specific readout of liquid refractive index and extinction coefficient by exploiting the features of wavefronts as information carriers. MHWF is constructed from the wavefronts of a focused vortex beam generated by a dielectric metasurface embedded in the liquid. The topological preservation of the vortex beam with orbital angular momentum ensures the integrity of the MHWF wavefront features, forming a unique three‐dimensional optical fingerprint. Specifically, the fingerprint carries wavefront profile, focal length evolution, and spatial intensity distribution information governed by the complex dielectric response of the liquid, which are deciphered using beam quality spatial variance, hyperbolic geometric propagation, and Fresnel diffraction theory. Furthermore, MHWF enables high‐sensitivity refractive index sensing with multi‐response. This work establishes an ultra‐compact optical metrology platform, paving the way for light‐matter interaction studies in liquid‐state photonics.

## Graphical abstract

A metasurface‐enabled helical wavefront fingerprint (MHWF) paradigm is proposed for quantitative and specific retrieval of a liquid medium's refractive index and extinction coefficient. The liquid's complex dielectric response governs the wavefront evolution of the metasurface‐generated focused vortex beam, forming a distinctive three‐dimensional optical field. The MHWF paradigm establishes an ultracompact, high‐precision platform for studying light‐matter interactions in liquid photonics.

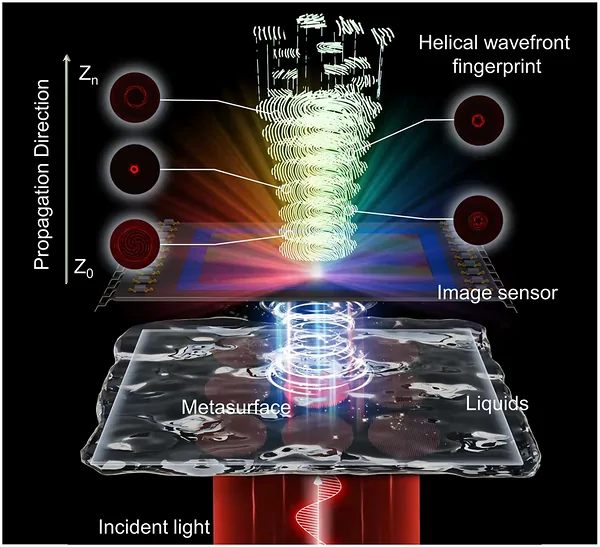

## 1 Introduction

Liquids, as core substances in nature, participate extensively in multi‐scale physical and chemical processes [1] involving molecular transport, heat transfer, and reaction dynamics. From a microscopic physical perspective, the macroscopic functions of liquids arise from molecular‐level intrinsic properties. The dielectric response of molecular systems to electromagnetic fields defines liquid optical properties, which are parameterized by optical constants. Optical properties fundamentally dictate light propagation, scattering, and attenuation in liquids [2, 3]. Furthermore, variations in molecular composition, concentration, and intermolecular interactions modulate refractive index and extinction coefficient in liquids. Specifically, variations in refractive index can reveal biomolecular concentration, reaction kinetics, and environmental variations, while the extinction coefficient directly reflects absorption and energy dissipation processes. Therefore, optical constants lay the physical basis for biological sensing [4, 5], chemical analysis [6, 7], and precision photolithography [8, 9]. Accurate measurement of liquid optical constants is a prerequisite for quantitative optical characterization and precise control of optical response in liquids. Currently, liquid optical constants are measured by refractometry and spectroscopic ellipsometry. Abbe refractometer, as a conventional optical device, measures the refractive index at a single wavelength [10]. Moreover, because its measurement principle does not quantify propagation‐induced optical attenuation within the medium, it cannot directly determine the extinction coefficient [11]. This limits the information content available for optical characterization and leads to degeneracy in liquids exhibiting identical refractive indices despite distinct electromagnetic responses [12]. Spectroscopic ellipsometry [13], by contrast, offers exceptionally high accuracy in retrieving optical parameters [14, 15], yet its reliance on costly, large‐scale instruments and complex calibration procedures limits its potential for miniaturization and on‐chip integration [16]. More critically, spectroscopic techniques that rely on external light incident on liquid surfaces inevitably suffer from gas‐liquid meniscus formation arising from surface‐tension‐driven curvature [17, 18], which depends on the liquid's wettability and contact angle under gravity [19, 20]. These menisci induce beam distortion and phase errors, which degrade measurement fidelity and ultimately compromise the accuracy of quantitative optical measurements in liquids [21].

An effective solution requires compact optical platforms capable of operating directly within liquids, enabling information‐rich mapping of light‐matter interactions onto the liquid's intrinsic optical response. Metasurfaces [16], subwavelength nanostructured arrays, enable versatile manipulation of electromagnetic waves and support advanced photonic applications in sensing [22, 23], holography [24, 25], radar [26, 27], lenses [28, 29], and imaging [30, 31]. Metasurface‐enabled strategies exploiting wavefront sensing [32, 33] or structural color responses [34] have been reported for liquid recognition. However, such approaches typically rely on large, task‐specific datasets and functions essentially as a statistical learning mapping, without providing access to intrinsic optical properties governed by the fundamental laws of light‐matter interaction in liquids [35, 36]. Fundamentally, for liquid media exhibiting highly similar dielectric responses, the wavefront of a single fixed propagation position leads to strong similarity in phase, thereby fundamentally limiting their discriminability [37]. Therefore, a metasurface platform capable of direct recording of axial optical field evolution is required to achieve quantitative characterization of intrinsic liquid optical constants, while avoiding dependence on large training datasets or complex optical instrumentation and enabling stable operation in sealed or microfluidic environments.

In this work, we propose and demonstrate a metasurface‐enabled helical wavefront fingerprint (MHWF) optical metrology paradigm for quantification of optical constants and refractive index sensing. The metasurface‐generated optical fingerprint encodes the light‐matter interaction through its wavefront profile, spatial intensity distribution, and focal length evolution, providing a physically interpretable and specific signature that reflects the intrinsic optical properties of the liquid. Beam Quality Spatial Variance (BQSV), Hyperbolic Geometric Propagation (HGP) model, and Fresnel diffraction theory are employed to decode optical field evolution so as to enable simultaneous retrieval of refractive index (*n*) and extinction coefficient (*k*). To our knowledge, prior explorations have not ventured into metasurfaces for the physically quantitative characterization of optical constants of media. Our findings suggest that MHWF enables high‐fidelity mapping between measured optical signals and material intrinsic optical properties of media, which points toward a new paradigm for compact and high‐precision optical metrology.

## 2 Results and Discussion

### 2.1 Concept of Metasurface‐Enabled Helical Wavefront Fingerprint

**FIGURE 1:**
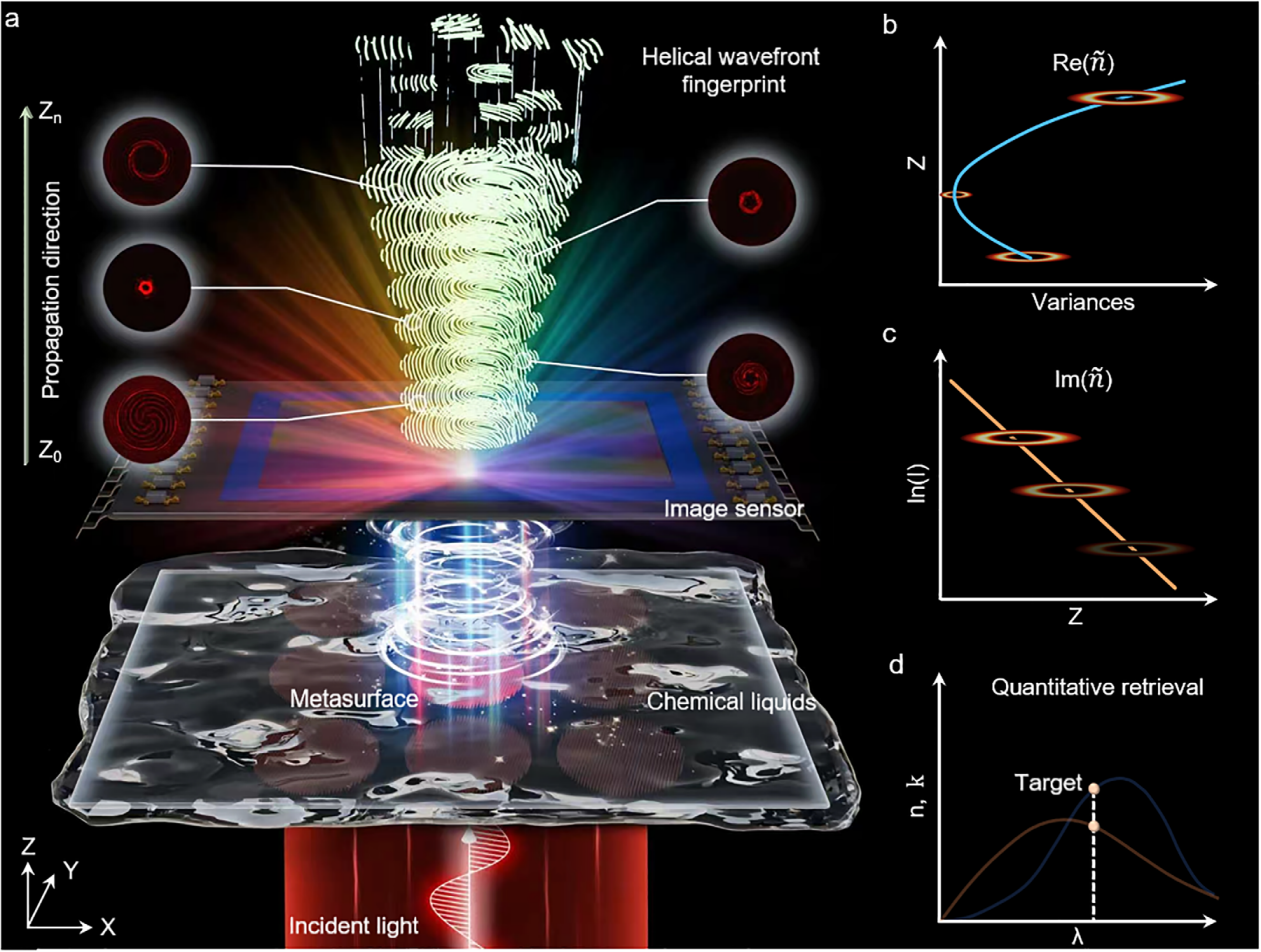
The conceptual illustration of the MHWF optical metrology paradigm. (a) The dielectric metasurface is fully embedded in the liquid medium. A linearly polarized incident beam (637 nm) is converted into a focused vortex beam. A series of wavefront distributions capturing the axial evolution of the optical field governed by the liquid's dielectric response is recorded using a CMOS camera. (b) Quantification of the liquid refractive index (*n*) by BQSV extraction of its propagation induced focal length evolution. (c) Quantification of the liquid absorption coefficient (*α*) by logarithmic fitting of its propagation‐induced intensity attenuation profile. (d) Quantitative retrieval of the real and imaginary parts of liquid optical constants at the target wavelength.

Accurate measurement of liquid optical constants is essential for understanding light‐matter interactions and optical response. From the perspective of electromagnetic theory, the interaction between light and liquid media is governed by their intrinsic dielectric response, which is compactly described by the complex refractive index that encodes both dispersion and absorption characteristics of the liquid (Section S1, Supporting Information). To establish a quantitative characterization and liquid‐specific linkage between the response of liquids and experimentally accessible optical signature, Figure 1 illustrates the conceptual principle of the metasurface‐enabled helical wavefront fingerprint (MHWF) metrology framework. The dielectric metasurface is fully embedded in the liquid medium and illuminated by linearly polarized light (637 nm), generating a focused vortex beam whose axial evolution and wavefront profile are governed by the liquid's dielectric response. A series of optical field images are recorded using a CMOS camera at multiple fixed positions (denoted as Z_1_, Z_2_, …, Z_n_) along the propagation optical axis in the liquid medium. MHWF constitute medium‐specific three‐dimensional optical field signatures formed by integrating wavefront distributions at different axial positions, in which the helical wavefront evolution reflects the liquid's intrinsic refractive index (*n*) and absorption coefficient (*α*) determined by its molecular composition. By recording the optical field evolution entirely within the liquid, our proposed metrology paradigm fundamentally avoids gas‐liquid interface interactions and establishes a novel alternative to conventional refractometry and ellipsometry. Meanwhile, the topological preservation of the vortex beam [38, 39] with orbital angular momentum ensures the integrity of the MHWF wavefront features, enabling a unique three‐dimensional optical fingerprint. The fingerprint encapsulates the modulation of the light field by the surrounding liquid medium. Variations in the *n* and *α* among different liquids induce slight shifts in the focal position and distinct intensity attenuation behaviors, thereby giving rise to characteristic differences in the optical field morphology. Specifically, the real part (*n*) of the liquid's complex refractive index is retrieved from the focal plane position, which is determined by the spatial variance of the beam spot profile along the MHWF (Figure 1b). Simultaneously, the imaginary part (*k*, *k* = *αλ*/4/π) of the complex refractive index is quantified by logarithmic fitting of the intensity attenuation of the MHWF, as shown in Figure 1c. For different pure liquids, this metrology method enables in situ reconstruction of liquid‐specific helical wavefronts, decoupling phase‐driven focusing from absorption‐driven attenuation to achieve direct mapping from unique three‐dimensional vortex field fingerprints to liquid optical constants at the target wavelength (Figure 1d).

**FIGURE 2:**
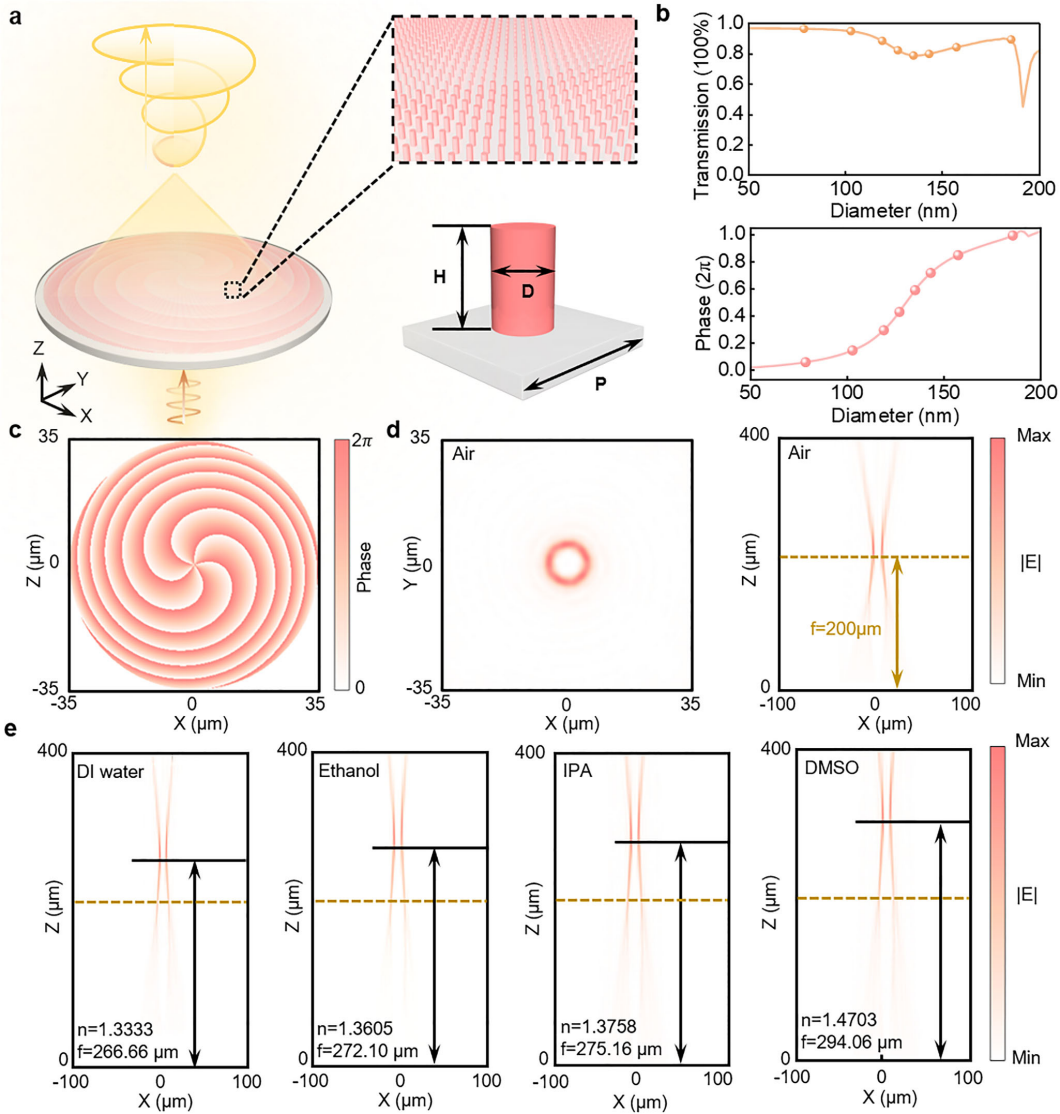
Design principles and numerical modeling of metasurfaces. (a) Proposed a‐Si:H nanopillar meta‐atom with a fixed height (H) and period (P) but with various diameters (D) on a silica substrate. (b) Transmission and phase pertaining to the chosen eight‐level meta‐atoms. (c) Calculated relative phase distribution of the designed metasurface to produce a vortex beam. (d) Electric‐field distributions for normal incidence in the *xz* and *xy*‐plane. Metasurface with a designed focal length of 200 µm. (e) Differences in the focal planes of vortex beams arising from variations in *n* of different liquid media.

To realize this metrology paradigm on a compact platform, a metasurface capable of simultaneously enabling strong light‐matter interaction and efficient structured‐light generation is required. Accordingly, we design ultrathin nanopillars as meta‐atoms and conduct rigorous analysis of their structural characteristics, far–field responses, and coupling interactions with the surrounding medium. Figure 2a illustrates a representative a‐Si:H nanopillar meta‐atom constituting the designed metasurface under incident light. The metasurface is composed of nanopillars arranged in a square lattice with a period (P) of 240 nm and a fixed height (H) of 300 nm placed on the silica substrate. The geometric diameter of the meta‐atoms dictates the phase modulation that generates focused vortex beams. The diameter (D) is varied to tailor the phase response, and the aspect ratio in the range of 1.6–3.9 ensures structural robustness and fabrication feasibility. The structural parameters of the meta‐atoms are determined using finite‐difference time‐domain (FDTD) simulations to achieve a full 2π phase shift with high transmittance (Figure 2b). The layout of the metasurface with an encoded phase gradient profile that renders the focused vortex beams is illustrated in Figure 2c. The desired phase profile is expressed as follows [32]:

(1) $$\varphi \left(x,y\right)=\frac{2\pi n}{\lambda }\left(f-\sqrt{x^{2}+y^{2}+f^{2}}\right)+l_{0}\tan^{-1}\left(\frac{y}{x}\right)$$

where (*x, y*) represents the coordinates corresponding to the center of each meta‐atom in the middle of the device, *l_0_* is the topological charge, and *f* is the focal length. According to Snell's law, the focal length of the vortex beam is determined by the desired local phase delay induced by the meta‐atoms and surrounding materials. Theoretically, the focal length of the metasurface‐generated vortex beam increases with the *n* of the surrounding medium. Furthermore, the Beer–Lambert law characterizes the attenuation of light intensity during propagation and establishes a theoretical framework for correlating propagation losses (*α* or *k*) with the optical properties of the medium. Simulated electric‐field profiles in the *x*‐*z* and *x*‐*y* cross sections under normal incidence (air background) confirms the generation of a vortex focal spot exhibiting a characteristic doughnut intensity distribution at a focal distance of 200 µm (Figure 2d). The focal‐plane beam size increases linearly with the topological charge *l_0_*_._ Therefore, from the perspective of in situ liquid characterization based on wavefront perception, a vortex beam with a topological charge of 5 is required to generate a relatively large spot size, thereby enhancing the discriminability of the optical field distribution and the significance of differences between liquids. The strong medium sensitivity of metasurface‐generated vortex beam enables them to act as unique optical fingerprints for liquids.

We theoretically analyze the dependence of vortex‐beam focal length and electric field distributions on the refractive index (RI) of four representative liquids, including deionized water (DI water), ethanol, isopropanol (IPA), and dimethyl sulfoxide (DMSO), as shown in Figure 2e. The focal length of the vortex depends on the *n* of the medium. The simulated focal length of the vortex beam increases with increasing *n* of the liquid substance. To demonstrate that the proposed framework probes intrinsic optical properties rather than superficial RI contrast, thereby creating physically test cases governed by distinct molecular‐level optical responses.

### 2.2 Experimental Characterization of Wavefront Evolution

**FIGURE 3:**
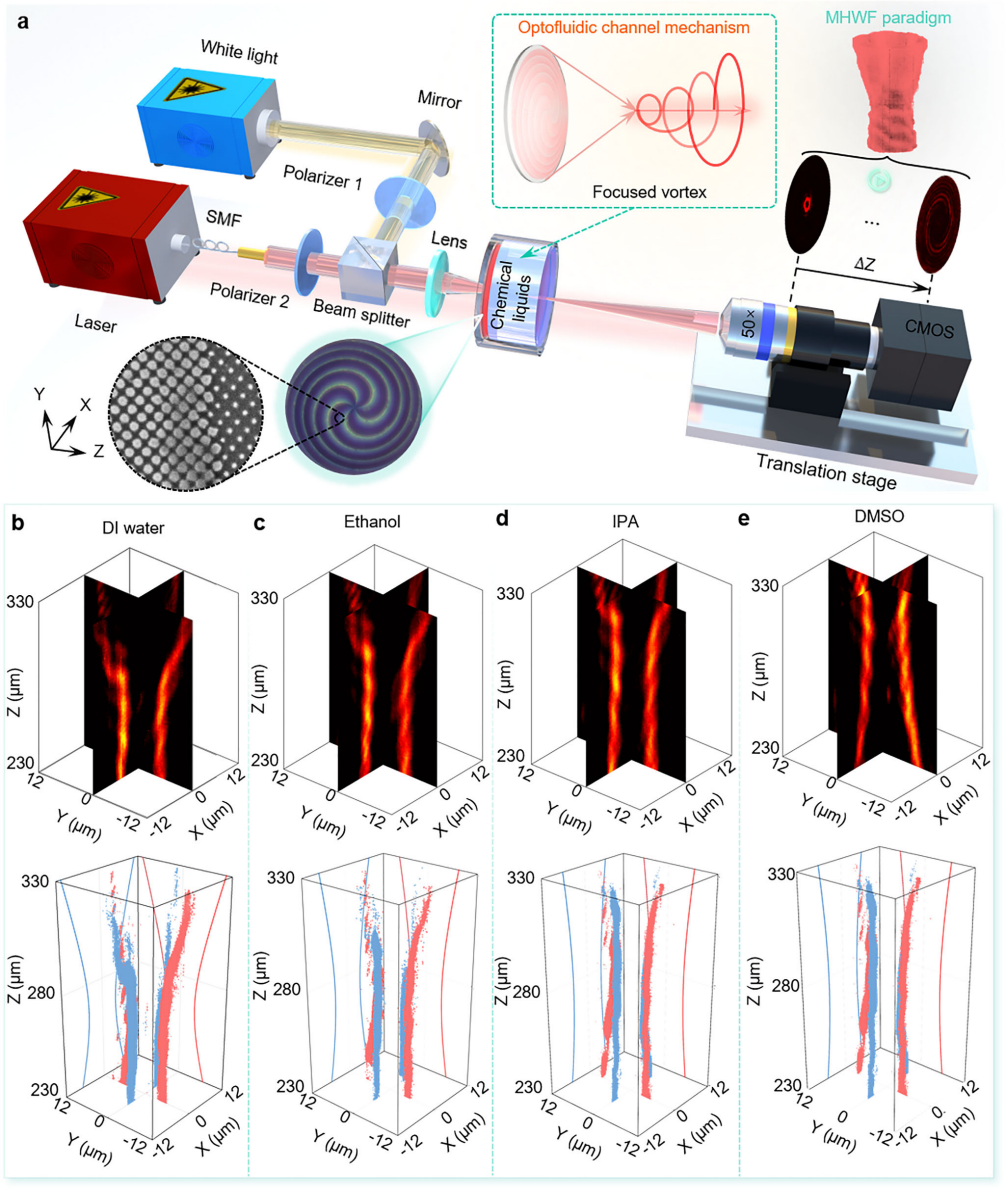
Test setup and specific MHWFs for different media. (a) Experimental setup for acquiring optical field distribution images in different liquid media. *xz* and *yz* cross‐sectional light field slices and hyperbolic model fitting of MHWFs of (b) DI water, (c)ethanol, (d) IPA, and (e) DMSO.

Following a series of rigorous simulations, the proposed metasurface is fabricated by electron beam lithography, as illustrated in Figure S1 and detailed in the Methods. Scanning electron microscopy images of high‐aspect‐ratio nanopillars with vertical sidewalls are displayed in Figure S2. To verify that MHWF faithfully encodes light‐matter interactions, the experimental setup is shown in Figure 3a, and further details are described in the Methods section. A white‐light path is employed for precise alignment the metasurface, camera, lenses, and other optical components. During the experiment, the white‐light source is turned off to avoid stray‐light interference, while the laser is performed to measure MHWFs of different liquid analytes. The optical constants of the medium modulate the optical field distribution, driving a continuous evolution of the focal position, optical spot morphology, and intensity distribution. Experimental characterization of optical field evolution along the optical axis in air is shown in Figure S3 of Section S4 of the supporting information. To visually illustrate the spatial evolution of the focused vortex beam propagating in the liquid, Figure 3b–e presents the orthogonal *x‐z* and *y‐z* cross‐sectional optical field slices extracted from MHWFs (Section S5, Figure S4, Supporting Information) obtained in DI water, ethanol, IPA, and DMSO liquid media. A propagation distance of 230–330 µm from the metasurface (Z_0_) is selected, which is sufficient to span the RI range of most liquids. Vortex light undergoes phase accumulation dominated by the medium's real refractive index *n*. Consequently, a higher *n* causes the focal plane position to be farther from the designed Z_0_ position.

Furthermore, the two orthogonal section optical field profiles are fitted with the HGP model (Section S6, Table S1, Supporting Information) to capture the optical behavior of focusing and subsequent divergence. Simultaneously, we define the Focus Ellipticity (FE) as the relative difference between the semi‐major axis lengths of the hyperbola along two perpendicular directions to evaluate and verify the robustness of our measurement setup. As shown in Figure S5, the FE values for the four liquids fluctuate but consistently remain below 12%, demonstrating the difference between the two real semi‐axes is much smaller than their average. The focal spot is only slightly elliptical, indicating good optical‐axis alignment and deviations that fall within normal experimental variation.

### 2.3 Quantitative Retrieval of Liquid Refractive Index

**FIGURE 4:**
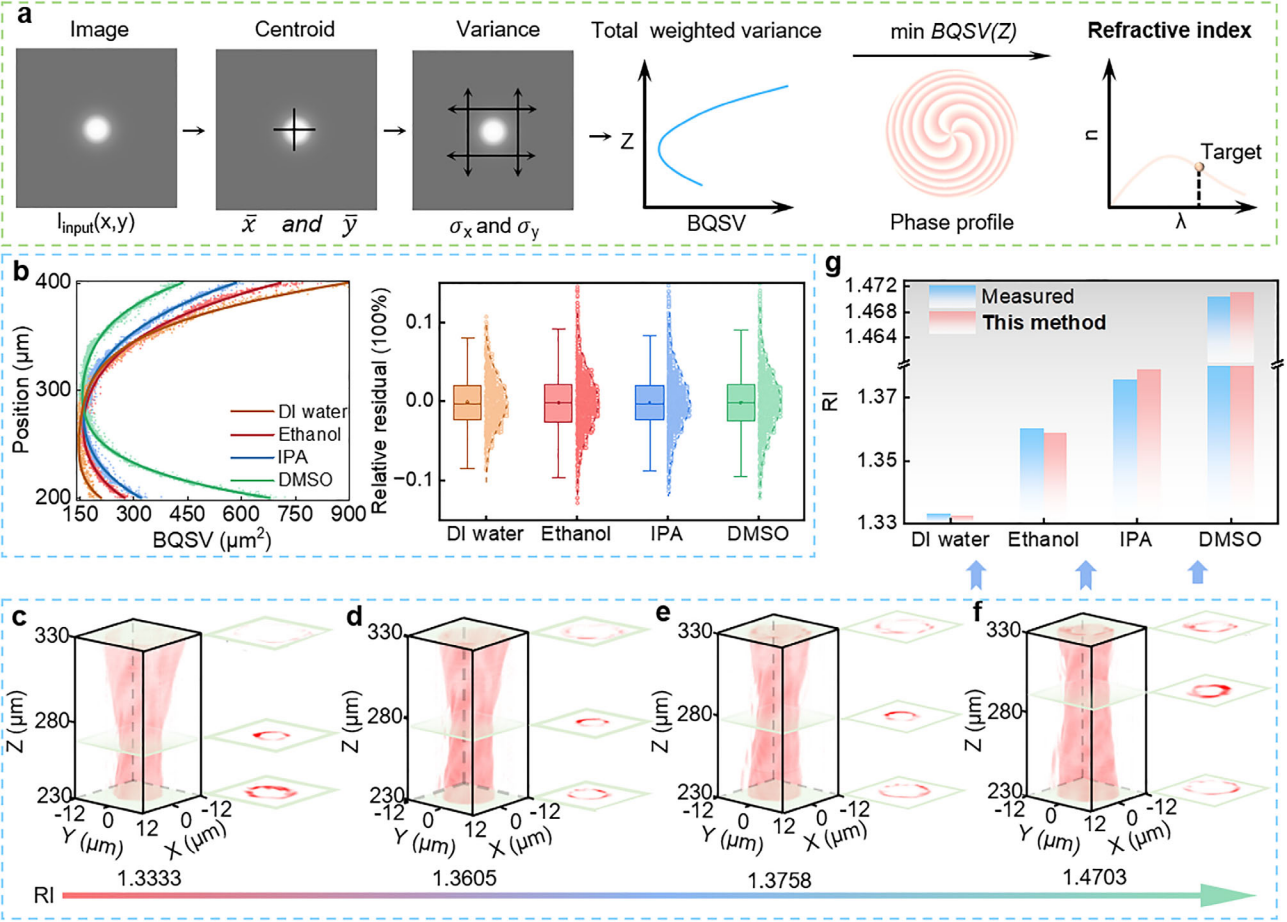
Experimentally measured MHWFs in various liquid media for quantitative characterization of liquid *n*. (a) Conceptual workflow for quantitative retrieval liquid *n* via BQSV. (b) BQSV with fitted curves and corresponding residual box plots derived from MHWF for different liquid media over a propagation range of 200–400 µm. (c–f) Experimental measurements of MHWFs for various liquids. (g) RI results obtained using the BQSV method and refractometer.

Within a stable and precision measurement setup, the optical field images acquired at different axial positions encode the liquid‐specific optical field through the combined evolution of phase, intensity distribution, and focal length. For the experimentally acquired optical field images, we first apply a denoising procedure to suppress noise arising from CMOS sensor dark current and ambient‐light scattering, as detailed in Section S7 of the Supporting Information. Furthermore, a detailed analysis of the denoised optical field distributions at each axial position constituting MHWF is required. As shown in Figure 4a, inspired by concepts of the second central moment and beam intensity profiles [40], we construct BQSV to quantitatively characterize the lateral geometric evolution of the light spot, with the theory provided in Section S8 of the Supporting Information. First, we determine the centroid (*x̄, ȳ*) of the intensity distribution from the optical field image *I*_input_ (*x, y*) with 3480 × 2160 pixels of the propagating vortex beam at a given axial position Z. After obtaining the centroid position, the deviation of each pixel from the centroid in the *x* and *y* orthogonal directions is calculated. Subsequently, we square the deviation of each pixel to amplify the influence of regions far from the center on the overall spot expansion behavior, and multiply this result by the pixel's intensity to obtain its contribution to the spatial variance in both directions. Finally, the sum of the BQSV (denoted as σ*_x_* and σ*_y_*, respectively) in the two orthogonal directions *x* and *y* of each optical field distribution image is evaluated to characterize its relationship with the axial position. Mathematically, aligning the centroid eliminates sensitivity to translational shifts of the spot, extracting the geometric second moment removes dependence on global intensity variations, and applying energy weighting suppresses the contribution of background noise. Consequently, the profile of the beam spot can be stably and reliably extracted even in non‐ideal experimental environments.

To validate this principle, we specifically examine the consistency of the BQSV in the *x* and *y* directions for spots of identical size under different positions and intensities, as shown in Figure S7a,b. However, for spots of different sizes, the relative centroid offset at each pixel location systematically changes with the expansion or contraction of the spot. Outer pixels exhibit larger geometric offsets in larger spots and therefore contribute more strongly to the spatial weighting process, whereas pixels near the center contribute only minimally due to their small offsets, as shown in Figure S8a–c. This weighted summation of offset and intensity ensures that the final variance metric accurately reflects lateral variations in spot size, while remaining unaffected by spot‐position drift or overall brightness changes. Therefore, the BQSV is positively correlated with the lateral size of the light spot, providing a direct physical rationale for focused vortex beams as the detection carrier. In contrast to conventional unfocused orbital angular momentum (OAM) beams, focused vortex light exhibits a clear contraction‐divergence evolution during propagation, resulting in a clear and unique minimum value for its variance curve. This avoids the ambiguity and instability typically faced by unfocused OAM beam in size determination and axial positioning. Based on this mechanism, the BQSV curve corresponding to MHWF exhibits the minimum value, which naturally defines the geometric focal plane of the vortex beam. As the focusing position is extremely sensitive to medium‐induced phase accumulation, its axial shift follows a monotonic dependence on the real part of the RI. Therefore, by accurately locating the focal plane position corresponding to the minimum variance, the *n* of the liquid can be robustly and quantitatively obtained.

Within the theoretical framework, we perform numerical simulations of MHWFs of representative liquids, as shown in Figure S9, their corresponding BQSV exhibits a pronounced minimum (266, 272, 275, and 294 µm) along the optical axis, coinciding with the focal plane of the MHWF. To accurately resolve the focal position within the focused‐divergent evolution, the optical field is sampled at a constant speed with an effective axial interval of 5 µm/60, as shown in Figure S4. This densely sampled optical field dataset provides sufficient spatial resolution to fully capture the focusing and subsequent divergent evolution of the MHWF in the medium. In the demanding and environmentally sensitive conditions of actual experiments, the BQSV derived from the densely sampled optical field images is calculated and fitted with by a power‐law model, as shown in Figure 4b. For all liquids, the experimental data are closely distributed near the fitted curve throughout the propagation range, indicating that this evolution is not a random imaging effect. The axial optical field of the MHWF is highly sensitive to the intrinsic electromagnetic response of the medium, and this response can be effectively characterized by a power‐law scaling model (Section S10, Supporting Information). More importantly, the residual distribution is centered at zero and exhibits approximately symmetrical statistical characteristics, detailed residual calculations are provided in Figure S10 of the Supporting Information. Along the propagation direction, BQSV exhibits a nonlinear variation characterized by an initial decrease during focusing followed by an increase during divergence. This process produces a well‐defined axial minimum, which corresponds to the focusing plane of the MHWF and agrees closely with the simulation results. Consistently, the corresponding MHWFs demonstrate that increasing *n* leads to a progressive extension of the axial focusing region (Figure 4c–f). DI water with a low refractive index therefore has a small focal position. Especially for DMSO, the property of high *n* causes the focal length position to be significantly shifted to a more distant axial region, a trend consistently observed in both numerical simulations and experimental reconstructions. The physical mechanism can be attributed to the preservation of the topological structure of the vortex phase [41] of the focused vortex field during propagation in the liquid medium. Specifically, although the RI of the liquid changes the focal position, the intrinsic OAM carried per unit electromagnetic energy (Section S11, Figure S11, Supporting Information) and its corresponding optical field distribution remain stable during propagation, thereby ensuring the robust repeatability and distinguishability of the helical wavefront evolution and optical field images. Meanwhile, we extract representative optical field distributions at 230 µm, the focal plane, and at 330 µm beyond the focal point. The spots at these axial positions deviate from an ideal donut profile, with local intensity fluctuations and imperfect ring shapes making single‐image morphology evaluation unreliable. In contrast, the BQSV proposed in this work captures only the lateral size variation of the light spot. Through energy‐weighted geometric statistics over all pixels, it inherently suppresses uncertainties arising from local morphological deviations. Figure 4 g summarizes the RI results derived from the focal‐length position using the BQSV method and compares them with the values provided by a commercial refractometer. The *n* extracted by the two methods exhibit excellent agreement for all four liquids, thereby validating the accuracy and reliability of *n* quantification enabled by the combination of the MHWF and BQSV framework.

### 2.4 Multi‐Linear Response of the MHWF for High‐Sensitivity RI Sensing

**FIGURE 5:**
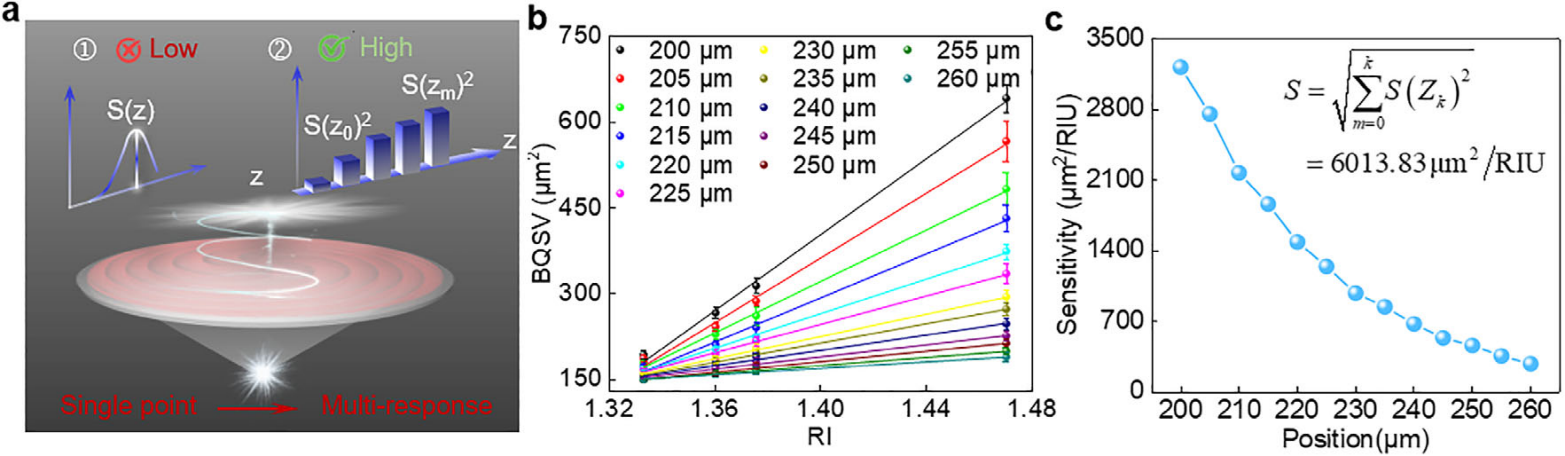
Multi‐linear response of the MHWF for sensing. (a) Conceptual diagram of MHWF for continuous multi‐response. (b) BQSV at different propagation positions for various media. (c) RI sensitivity under multi‐response obtained by MHWF.

Based on the quantitative retrieval of refractive index, analysis of each frame of the light‐field images forming the MHWF reveals high sensitivity to the optical properties of the medium, such that the values at the same axial position differ significantly. Continuous sampling along the propagation direction therefore constructs a high‐dimensional response space (Figure 5a), effectively amplifying and enhancing the resolvability of RI information. To elucidate the contribution of vortex phase to RI sensing, we define a sensitivity enhancement factor as the ratio of the RI sensitivity of the focused vortex beam within the MHWF framework to that of the focused Gaussian beam generated by a conventional metalens, as detailed in Section S12 of the Supporting Information. As shown in Figure S12e, the factor remains greater than unity at all propagation positions, further indicating that the vortex phase enhances the distinguishability of the axial BQSV trajectories by inducing more pronounced radial expansion of the optical field. Furthermore, the experimental results (Figure 5b) demonstrate a strong linear correlation between the RI and BQSV at different propagation positions (R^2^>0.99, Table S3, Supporting Information), arising from the progressive shrinkage of the spot size and the corresponding decrease in these values with increasing propagation distance. At a fixed propagation distance, a higher *n* modifies the axial evolution of wavefront curvature by altering the phase matching between propagation and the metasurface, thereby shifting the focal position backward. As a result, the spot becomes larger, and the corresponding BQSV increases at the same axial position. This indicates that the axial evolution of the MHWF is fundamentally governed by *n modulated* phase accumulation. Leveraging the multi‐linear response space of the MHWF, the sensitivity to RI changes is 6013.83 µm^2^ /RIU over a wide detection range of 1.3333 to 1.4703, as shown in Figure 5c.

### 2.5 Quantitative Retrieval of Liquid Extinction Coefficient

**FIGURE 6:**
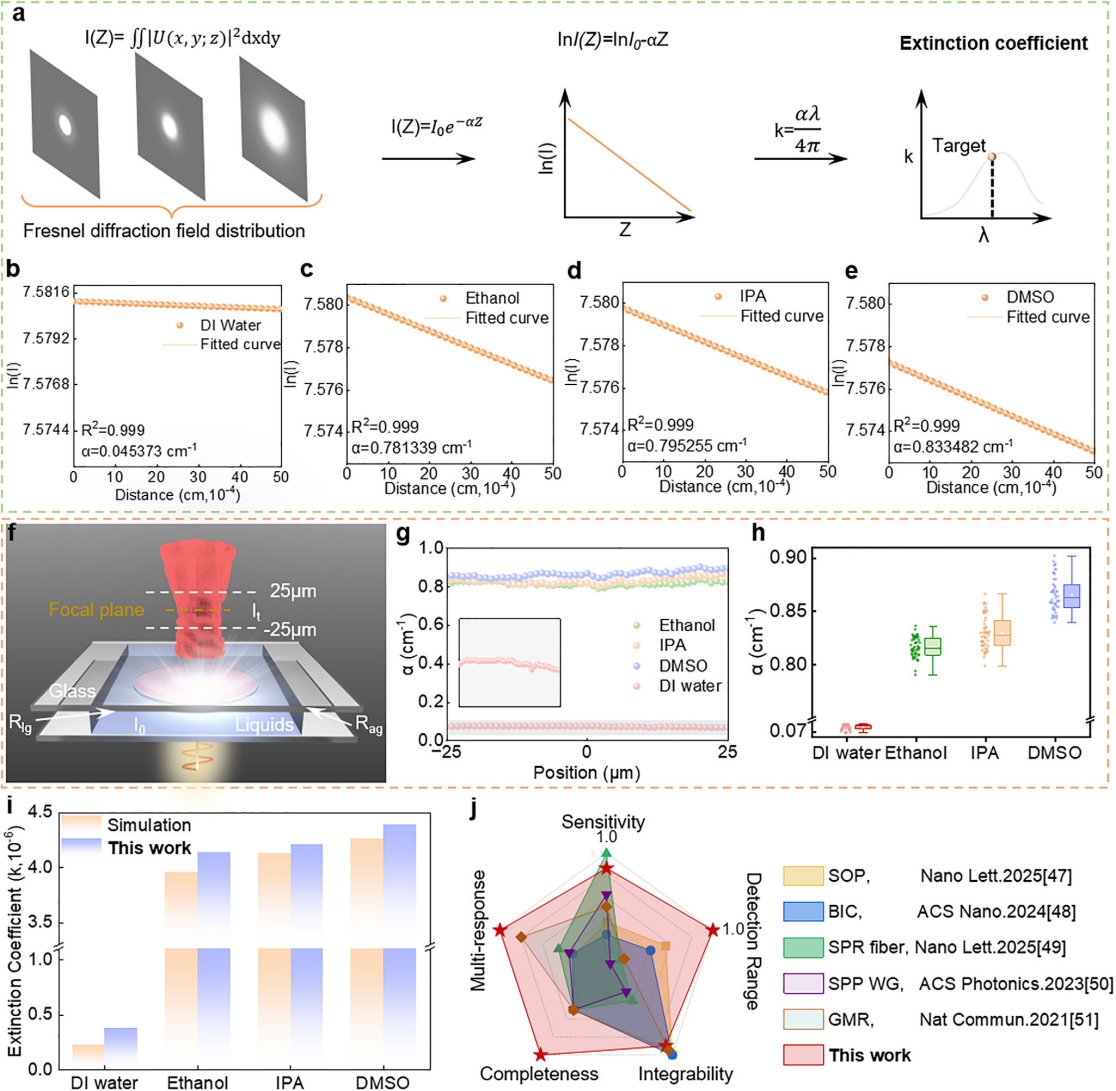
Experimentally measured MHWFs in various liquid media for quantitative characterization of liquid *k*. (a) Conceptual workflow for liquid *k* based on Fresnel diffraction theory and the Beer–Lambert law. (b–e) Simulation results of the linear between the logarithm of light intensity and propagation distance for different liquids.The slope corresponding to *α*. (f) Schematic illustration of experimental *k* retrieval based on the MHWF framework. (g) The *α* value extracted from different axial positions under a fixed optical path configuration. (h) Box plot statistics of *α* extracted from multiple axial positions in experiments. (i) Quantitative retrieval of the liquid *k* for simulated and experimental results. (j) Radar chart of different optical metrology platforms. SOP, State‐of‐Polarization. BIC, Bound state in the Continuum. SPR, Surface Plasmon Resonance. SPP, Surface Plasmon Polariton. WG, Waveguide. GMR, Guided Mode Resonance.

The extinction coefficient of highly transparent liquids in the visible range remains insufficiently studied, as their inherently weak absorption produces signals close to the detector noise floor, posing significant challenges for reliable experimental measurement. The *k* describes the intensity attenuation process of light propagating in a medium due to absorption and is characterized by the imaginary part of the complex refractive index. To ensure the reliability of *k* retrieval, we first verify the applicability of the scalar angular‐spectrum representation to the metasurface‐generated focused vortex field, as detailed in Section S14 of the Supporting Information. Full‐vector FDTD simulations and quantitative calculations show that the longitudinal electric‐field energy fraction is very low, indicating that the optical field is predominantly transverse and supporting the validity of the scalar approximation in the present weakly focused regime. This result is also consistent with previous studies in which the longitudinal electric‐field component is used to evaluate the validity of the scalar approximation [42, 43]. Furthermore, the *k* values retrieved using the Fresnel diffraction and the angular‐spectrum exhibit good agreement. The Fresnel diffraction approximation also offers clear advantages in computational speed and memory efficiency. Accordingly, we adopt the Fresnel diffraction approximation model to characterize optical field complex amplitude *U* (*x, y; z*) evolution and simulate the propagation of metasurface‐generated focused vortex beam in a medium, as shown in Figure 6a. In this theoretical framework, the propagation of the light field can be regarded as the combined action of free‐space diffraction operators together with medium‐induced phase and absorption operators. Therefore, the Fresnel diffraction integral [44] can naturally embed the exponential decay term caused by absorption into the propagation kernel, thus uniformly describing the diffraction, phase accumulation, and intensity decay of light in a single expression. Based on this, for a optical field propagating in a liquid with a complex refractive index $\tilde{n}$ *= n+ik*, its Fresnel diffraction integral can be written as:

(2) $$\begin{array}{cc} U(x,y;z) & =\frac{e^{ik_{0}\left(n+ik\right)z}}{i\lambda_{0}z}\;\int \int \;U_{0}\left(x^{\prime },y^{\prime }\right)\\ & \;\exp\left[\frac{ik_{0}\left(n+ik\right)}{2z}\left({\left(x-x^{\prime }\right)}^{2}+{\left(y-y^{\prime }\right)}^{2}\right)\right]dx^{\prime }dy^{\prime } \end{array}$$

where *k_0_ = 2π/λ_0_* is the vacuum wavenumber. The real part *n* controls phase accumulation, thereby controlling the evolution of the focal point, while the imaginary part *k* leads to exponential decay conforming to the Beer–Lambert law. Then, the two‐dimensional intensity cross‐section at each propagation position Z is obtained from this diffraction theory. Integrating the transverse intensity distribution over the area yields the total intensity, as follows:

(3) $$I\left(z\right)=\;\int \int \;|U\left(x,y;z\right){|}^{2}dxdy$$

The optical field propagating in an absorbing medium exhibits an exponential decay in intensity with propagation distance, and the overall integrated intensity follows the Beer–Lambert law [45]. By taking the logarithm of the integrated intensity and performing linear fitting, the *α* can be extracted. Finally, based on the physical relation between light absorption and the imaginary part of RI, the extinction coefficient *k* of the liquid at a specific wavelength can be calculated. This method eliminates the need for sophisticated instrumentation and relies solely on the energy attenuation of the three‐dimensional field distribution to achieve quantitative retrieval of the imaginary RI of the liquid. With the theoretical framework, Figure 6b–e show that logarithmic light intensity exhibits a linear dependence on propagation distance for focused vortex beam propagation within 25 µm before and after the focal plane of the light field in different liquid chemicals. The favorable linearity (R^2^ > 0.99) confirms that the Beer–Lambert law effectively characterizes the attenuation behavior of light during propagation caused by the inherent absorption of the medium.

In the experiment, the optical path difference of the beam propagating through the liquid remains unaltered by the microfluidic configuration, resulting in a consistent optical field intensity. Meanwhile, the focusing and diverging behavior of the beam leads to subtle variations in local optical intensity that remain below the sensitivity threshold of the camera detector. Therefore, the intensity is further analyzed within plus‐minus 25 µm around the focal plane for different MHWFs, as shown in Figure 6f. As the beam passes through four interfaces sequentially during propagation, Fresnel reflection loss occurs at each interface. Furthermore, the optical intensity is attenuated by intrinsic absorption as it propagates through the test liquid. Therefore, the output light intensity recorded by the camera can be expressed as:

(4) $$\begin{array}{cc} I_{t}=I_{0}\left(1-R_{ag}\right)\left(1-R_{lg}\right)e^{-\alpha L} & =I_{0}\left(1-{\left(\frac{n_{a}-n_{g}}{n_{a}+n_{g}}\right)}^{2}\right)\\ & \;\times \left(1-{\left(\frac{n_{l}-n_{g}}{n_{l}+n_{g}}\right)}^{2}\right)e^{-\alpha L} \end{array}$$

where *I_0_* is the incident light intensity through metasurface, *R_ag_* and *R_lg_* are the reflectances at the air/glass and glass/liquid interfaces, respectively. *n_a_*, *n_g_* and *n_l_* represents *n* of air, liquid and glass, respectively. Accordingly, Figure 6g shows the consistent *α* obtained from the output optical intensities measured at different axial positions. The statistical distribution of the *α* extracted from DI water, ethanol, IPA, and DMSO, as shown in Figure 6h. The *α* values are tightly clustered, and the close agreement between the mean and median indicates strong robustness of the method, while the obvious separation between different liquids verifies the method's sensitivity to inherent absorption differences. The *k* for each liquid medium is highly consistent with the simulation results (Figure 6i). The proposed quantitative inversion of *k* based on MHWF fundamentally bypasses the traditional cumbersome and complex optical path tuning devices [46], establishing a new metrology paradigm with simplified architecture and robustness. Figure 6j summarizes representative and recent paradigms for optical constants characterization, including nanograting‐based state‐of‐polarization [47], metasurface‐based bound states in the continuum [48], fiber‐based surface plasmon resonance [49], waveguide‐based surface plasmon polariton mode [50], and nanohole‐array based guided‐mode resonance [51]. However, these approaches cannot achieve extinction coefficient measurement and typically excel only in one or a few performance dimensions, limiting their application as general‐purpose optical metrology tools across various scenarios (Section S15, Supporting Information). In contrast, the MHWF theory proposed here simultaneously realizes a wide detection range, high sensitivity, and the retrieval of *n* and *k* on a compact metasurface platform, representing a new paradigm for lightweight and high precision optical metrology. Furthermore, the specific mapping established between MHWF and chemical liquids enables rapid identification and quantitative characterization of unknown pure liquids.

## 3 Conclusion

In summary, we demonstrate a proof‐of‐concept for quantitative retrieval and analysis of liquid optical constants via a single‐layer metasurface to generate a focused vortex beam with a predefined topological charge. The wavefront characteristics of the beam in a liquid medium are governed by the liquid's complex refractive index, resulting in a specific optical fingerprint that enables MHWF to serve as a macroscopic mapping of the liquid's microscopic optical parameters. Furthermore, the proposed MHWF constructs a multi‐response linear space, we characterize BQSV as a function of RI and demonstrate an RI sensor with a 6013.83 µm^2^/RIU sensitivity over a wide detection range of 1.3333–1.4703. Indeed, the MHWF concept establishes a new physical framework for characterizing light‐matter interactions and provides a generalizable route toward quantitative retrieval of optical constants and material identification across gases and other material systems.

## 4 Experimental Methods

### 4.1 Metasurface Fabrication Process

The proposed metasurface was fabricated on a 1mm‐thick quartz substrate, as depicted in Figure S2. Before the fabrication process, the substrate was cleaned in three consecutive cycles, each consisting of acetone, isopropyl alcohol, and deionized water to enhance its adhesion to the a‐Si:H film. After a 300 nm‐thick a‐Si:H film was deposited on the substrate using plasma‐enhanced chemical vapor deposition (Plasmalab 100, Oxford). A positive electron beam resist (ZEP 520A, Zeon Chemical) was spin‐coated on the substrate. Additionally, a layer of Espacer (300Z, Showa Denko) was subsequently coated to prevent the electron beam from charge accumulation during exposure process. The resist patterning was conducted by electron beam lithography system (ELS‐Boden 125, Japan). Subsequently, the development was performed by immersing the samples in developer (ZED‐N50, Zeon Chemical). To fabricate the critical mask required for further processing, a 60nm‐thick aluminum (Al) film was deposited on the substrate by electron beam evaporation (Temescal BJD‐2000, America), followed by a lift‐off process using a ZEP remover (ZDMAC, Zeon Co.) to remove the photoresist and transfer the pattern to the aluminum layer. The patterned Al was used as a hard mask during dry etching to transfer the designed pattern onto the underlying a‐Si:H layer by Inductively coupled plasma reactive ion etching (OxfordPlasmalab System l00, UK), with the etching conditions optimized by controlling the mixture of SF_6_ and CHF_3_ gases. This optimization created nanopillars characterized by a high aspect ratio and vertical sidewalls. Finally, the Al layer was removed by Al wet etchant and the metasurface processing was completed.

### 4.2 Measurement Setup for Metasurface Characterization

The SEM images were measured by a field‐emission scanning electron microscope (Sigma‐HD, ZEISS). The used accelerating voltage is 5 kV, and the working distance is kept at 4.5 mm. To ensure complete contact between the liquid and the metasurface, the liquids were injected into a polydimethylsiloxane (PDMS) fluidic channel equipped with a microscope cover glass window. The developed metasurface was evaluated using a home‐made setup (Figure 3a). The laser beam generated by the laser source (FISBA READYSpot 488) was collimated by an optical fiber collimator, polarization‐selected by a linear polarizer, and focused onto the metasurface through a focusing lens. The magnified image of the focal spot was captured using an industrial CMOS camera (X5CAM4K8MPB, 3840 × 2160 pixels, 60 fps) equipped with objective lenses (50×, NA = 0.42, Plan Apo NIR). A high‐precision motorized stage carrying the camera was moved along the *z*‐axis at 5 µm/s to capture the optical field characteristics.

### 4.3 Numerical Simulation

The transmission spectrum and phase responses of the meta‐atoms were calculated by the commercial software Lumerical FDTD Solutions. The periodic condition was used along the *x* and *y*‐direction. Perfectly matched layers were applied in the transmission and reflection directions to absorb the outgoing waves. The RI of a‐Si:H was taken from the material date of the software. The RI of silica was fixed at 1.444. A MATLAB code based on the designed phase profile was used to calculate and output the pattern structure of the metasurface. The focused vortex beam and electric‐field distribution generated by the metasurface were simulated using the commercial Lumerical FDTD solver with perfectly matched layer boundary conditions.

## Author Contributions

**Yuting Zhou**: conceptualization, methodology, Writing – original draft, Writing – review and editing, formal analysis, investigation, visualization. **Duk‐Yong Choi**: resources, writing – review and editing. **Sang‐Shin Lee**: writing – review and editing, validation. **Feiyu Jiao**: project administration, visualization. **Xun Guan**: writing – review and editing, funding acquisition, resources, supervision. **Yansong Du**: software, visualization. **Simeng Li**: formal analysis, software, data curation. **Hongliang Li**: writing – review and editing, conceptualization, supervision, funding acquisition, validation. **Fei Ding**: writing – review and editing, validation, investigation. **Xuechao Yu**: writing – review and editing, validation, supervision, funding acquisition. **Jin Tae Kim**: writing – review and editing.

## Conflicts of Interest

The authors declare no conflicts of interest.

## Supporting information




**Supporting File**: advs77099‐sup‐0001‐SuppMat.docx.

## Data Availability

The data that support the findings of this study are available from the corresponding author upon reasonable request.
