# Supplementary material for: Metasurface‐Enabled Helical Wavefront Fingerprint Metrology for Quantitative Retrieval of Liquid Optical Constants
Source: Adv Sci (Weinh). 2026 Aug 24:e77099. Online ahead of print. doi: 10.1002/advs.77099 (PMC13502272; doi:10.1002/advs.77099)
Supplement: Supplementary file 1 [file ADVS-9999-e77099-s001.docx]

Supporting Information

**Metasurface-enabled helical wavefront fingerprint metrology for quantitative retrieval of liquid optical constants**

Yuting Zhou^1^, Hongliang Li^2,^*, Simeng Li^1^, Feiyu Jiao^1^, Yansong Du^1^, Jin-Tae Kim^3^, Sang-Shin Lee^4^, Duk-Yong Choi^5^, Fei Ding^6,^*, Xuechao Yu^2,^*, and Xun Guan^1,^*

**Affiliations:**

*^1^Tsinghua Shenzhen International Graduate School, Tsinghua University, Shenzhen 518055, China*

*^2^Key Laboratory of Multifunctional Nanomaterials and Smart Systems, Chinese Academy of Sciences, Suzhou Institute of Nano-Tech and Nano-Bionics, Suzhou 215123, China*

*^3^Quantum Technology Research Department, Electronics and Telecommunications Research Institute, Daejeon 34129, South Korea*

*^4^Department of Electronic Engineering, Kwangwoon University, Seoul 01897, South Korea*

*^5^ Department of Quantum Science and Technology, Research School of Physics, Australian National University, Canberra, ACT 2601, Australia*

*^6^ School of Electronic Science and Technology, Eastern Institute of Technology, Ningbo 315200, China*

*Correspondence should be addressed to H.L., F.D., X.Y. or X.G. (E-mails: hlli2025@sinano.ac.cn, [fding@eitech.edu.cn](mailto:fding@eitech.edu.cn), xcyu2022@sinano.ac.cn or xun.guan@sz.tsinghua.edu.cn)

Section S1: Electromagnetic origin of optical parameters

Section S2: Fabrication process for the metasurface

Section S3: Scanning electron microscope images of the fabricated metasurface

Section S4: Experimental characterization of the optical field evolution along the optical axis at

air

Section S5: Metasurface-enabled Helical Wavefront Fingerprints for probing light-matter interactions in liquid media.

Section S6: Hyperbolic Geometric Propagation model for the focusing and subsequent

divergence behaviour of the MHWF

Section S7: Denoising procedure for raw optical field images

Section S8: Mechanism of Beam Quality Spatial Variance characterizing the geometric evolution

of light spots

Section S9: Numerical simulations for MHWFs and corresponding BQSV curves

Section S10: The finite linear combination of power function characterizes the BQSV of MHWF

Section S11: Evolution of the transverse and longitudinal components of OAM along the z-axis.

Section S12: Comparison of focused vortex beam within the MHWF framework and focused Gaussian beam

Section S13: BQSV at different propagation positions for various medium and goodness of fit

Section S14: Validation of the scalar angular-spectrum representation for MHWF

Section S15: Potential applications of MHWF

**Section S1: Electromagnetic origin of optical constants**

The interaction between electromagnetic waves and matter is fundamentally described by the complex dielectric function^[1]^, as follows:

|  | $\varepsilon(\omega)=\varepsilon^{'}(\omega)+i\varepsilon^{''}(\omega)$ | (1) |
| --- | --- | --- |

where 𝜀′(𝜔) represents the energy-storage capability associated with molecular polarization, and 𝜀′′(𝜔) accounts for electromagnetic energy dissipation arising from absorption processes. These two quantities together constitute the intrinsic frequency-dependent electromagnetic response of a material. For non-magnetic media (*μ≈*1), which is a valid approximation for most liquids in the optical regime, the dielectric function is directly related to the complex refractive index, as follows:

|  | $\tilde{n}(\omega)=n(\omega)+ik(\omega)$ | (2) |
| --- | --- | --- |

Through the relation, as follows:

|  | $\tilde{n}^{2}(\omega)=\varepsilon(\omega)$ | (3) |
| --- | --- | --- |

Expanding this expression yields^[2, 3]^, as follows:

|  | $\left\{\begin{matrix} n^{2}(\omega)-k^{2}(\omega)=\varepsilon^{'}(\omega) \\ 2n(\omega)k(\omega)=\varepsilon^{''}(\omega) \end{matrix}\right.$ | (4) |
| --- | --- | --- |

These relations indicate that the refractive index $n$governs the phase velocity and phase accumulation of light propagating through the medium, while the extinction coefficient $k$ characterizes optical absorption and energy dissipation.

Importantly, the real and imaginary components of the optical response are not independent. They are fundamentally linked through the Kramers-Kronig relations^[4]^, which arise from the causality of light-matter interactions. For the complex refractive index, the Kramers-Kronig relations can be expressed as:

|  |  | (5) |
| --- | --- | --- |

where $\mathcal{P}$denotes the Cauchy principal value. These relations reflect the fact that optical dispersion and absorption originate from the same underlying electromagnetic processes and are causally connected across the entire frequency spectrum.

Consequently, simultaneous access to both 𝑛 and 𝑘 provides a physically consistent and complete description of the material’s electromagnetic response. In the context of the present work, the MHWFs encode phase accumulation governed by 𝑛 and intensity attenuation determined by 𝑘, enabling quantitative retrieval of both parameters within a unified, physically interpretable framework.

**Section S2: Fabrication process for the metasurface**

**Figure S1.** Schematic illustration of the fabrication process flow for the metasurface.

**Section S3: Scanning electron microscope images of the fabricated metasurface**

**Figure S2.** SEM images of the fabricated metasurface.

**Section S4: Experimental characterization of the optical field evolution along the optical axis at air**

A high-precision motorized stage carrying the camera was translated along the z-axis at 5 μm/s, enabling continuous acquisition of the optical field characteristics at successive axial positions along the optical path.

**Figure S3**. Optical field distributions at different optical axis positions (Z) ranging from 0 to 500 µm at λ = 637 nm.

**Section S5: Metasurface-enabled Helical Wavefront Fingerprints for probing light-matter interactions in liquid media**

The propagation field of vortex light with orbital angular momentum in a medium is simultaneously modulated by the phase accumulation governed by the refractive index *n* and the absorption governed by the extinction coefficient 𝑘. The dual modulation imprints liquid-dependent signatures onto the three-dimensional optical field, enabling it to function as a fingerprint signal that characterizes the intrinsic physical properties of the liquid. Illumination of the metasurface with linearly polarized light produces a focused vortex beam whose helical wavefront undergoes distinctly liquid-dependent evolution throughout propagation, as illustrated in Figure S4. The MHWF encode rich information about the intrinsic optical parameters of the liquid, encompassing wavefront morphology, intensity attenuation, and the evolution of the focal trajectory, all of which are intrinsically linked to the *n* and *k* of the liquid. Compared with traditional ellipsometers limited by beam deflection and spot distortion at the gas-liquid meniscus, metasurfaces leverage their ultrathinness, near-field capability, and compactness to establish direct contact with the liquid medium and the generation of stable and resolvable MHWFs within it. Harnessing these fingerprint wavefront features eliminates the need for spectral scanning or external interferometry, enabling traceable, interpretable multi-parameter quantification and precise liquid identification, thereby establishing an entirely new framework for liquid optical characterisation.

**Figure S4.** Four representative liquid MHWFs constructed from wavefronts measured 200-400μm downstream from the metasurface (Z₀) position.

**Section S6: Hyperbolic geometric propagation model for the focusing and subsequent divergence behaviour of the MHWF**

The focusing and later divergence of the MHWF exhibit optical characteristics that align well with the geometric features of a hyperbolic function. Leveraging this correspondence, we adopt a hyperbolic model to characterize the field evolution and extract physically interpretable parameters^[5]^. The optical behaviour of the MHWF is therefore described by the following expression:

|  | $x^{2}=pz^{2}+qz+r$ | (6) |
| --- | --- | --- |
|  | $a=\sqrt{r-\frac{q^{2}}{4p}}$ | (7) |

The coefficients *p, q,* and *r* are the quadratic fitting coefficients of the vortex trajectory and 𝑎 denote the semi-major axis of the fitted hyperbola. Within this framework, the optical field profiles in the orthogonal *xz-* and *yz*- cross-sections are modeled to capture the focusing and subsequent divergence behaviour dynamics, as shown in Figure 3. The coefficients and models for four representative liquids are listed in the Table S1.

**Table S1**: Fitting coefficients and model parameters for four representative liquids

| **Cross section** | X-Z | Y-Z |
| --- | --- | --- |
| **DI water** | $x^{2}=0.02372z^{2}-12.62z+1694$ | $y^{2}=0.02372z^{2}-12.62z+1695$ |
| **Ethanol** | $x^{2}=0.0115z^{2}-6.257z+866.2$ | $y^{2}=0.0115z^{2}-6.253z+868.7$ |
| **IPA** | $x^{2}=0.006762z^{2}-3.72z+530.2$ | $y^{2}=0.006758z^{2}-3.717z+532.2$ |
| **DMSO** | $x^{2}=0.01044z^{2}-6.138z+918.8$ | $y^{2}=0.01043z^{2}-6.133z+922.4$ |

Then, to evaluate the optical axis alignment and the stability of the test setup in the experiment, we define the focal ellipticity (FE), which is expressed as the relative difference in the semi-major axis lengths of the hyperbola along two perpendicular directions, as shown in the following formula:

|  | $\alpha_{focus}=\frac{\left\vert a_{x}-a_{y}\right\vert}{(a_{x}+a_{y})/2}\times100\%$ | (8) |
| --- | --- | --- |

where *a_x_* and *a_y_* represent the semi-major axis lengths of the hyperbolic model at the *xz* and *yz* sections, respectively. As shown in Figure S5, although minor fluctuations are observed, the FE values for all four liquids consistently remain below 12%. According to the FE definition, such low values indicate that the focal spot is nearly circular. This confirms excellent optical-axis alignment, with residual deviations well within the normal experimental tolerance. Consequently, the extracted MHWF parameters can be attributed primarily to the liquid-induced propagation behaviour rather than alignment artifacts, ensuring reliable and physically interpretable characterization.

**Figure S5**. FE values of four representative liquids.

**Section S7: Denoising procedure for raw optical field images**

In the experiments, optical field images are acquired in a dark environment established using an optical table and light-shielding cloth. Nevertheless, CMOS sensor dark current and ambient scattered light are still superimposed on the actual optical field distribution as background intensity, thereby introducing random noise. Therefore, raw optical field images are denoised before subsequent calculations. With a camera frame rate of 60 fps and a translation-stage speed of 5 μm/s, we acquire 2400 CMOS background images over a 200 μm propagation range with the laser switched off, while maintaining the same exposure time, gain, and environmental conditions as those used for the optical field measurements in the medium. Due to the uncertainty and randomness of the noise, the random noise in the optical field and background image is independent. Even when the two images have matching frame number, they do not correspond to the same noise realization, and frame-by-frame subtraction would introduce additional random fluctuations. Therefore, we average the 2400 background images pixel-by-pixel at the same pixel location to obtain a mean background noise distribution. Figure S6 illustrates the denoising process of the raw optical field images. First, multiple CMOS background images are averaged pixel-by-pixel at the same pixel location to obtain the mean background noise distribution, which is then subtracted from each raw optical field image. Finally, the BQSV values are calculated from the denoised optical field image.

**Figure S6**. Pixel-by-pixel denoising procedure of optical field images for BQSV calculation.

**Section S8: Mechanism of Beam Quality Spatial Variance characterizing the geometric evolution of light spots**

Quantitative characterization of beam evolution a is a central problem in optical field metrology and structured light analysis. Conventional metrics, such as beam width or peak intensity, often fail to capture the intrinsic geometric evolution of complex optical fields, particularly in the presence of non-uniform intensity distributions. To overcome this limitation, we introduce the Beam Quality Spatial Variance (BQSV) concept as a geometry-based descriptor of beam evolution. By weighting spatial coordinates with the local optical intensity^[6]^, BQSV jointly encodes the spatial distribution and energy localization of the light field, providing a robust, intensity and position-independent metric that directly reflects the lateral extent and geometric characteristics of structured beams. The theoretical formula as follows:

|  | $\begin{matrix} \bar{x}=\frac{\iint_{\Omega} x\vert I(x,y)\vert dxdy}{\iint_{\Omega} \vert I(x,y)\vert dxdy} \\ \bar{y}=\frac{\iint_{\Omega} y\vert I(x,y)\vert dxdy}{\iint_{\Omega} \vert I(x,y)\vert dxdy} \\ \sigma_{x}^{(I)}=\frac{\iint_{\Omega} (x-\bar{x})^{2}\vert I(x,y)\vert dxdy}{\iint_{\Omega} \vert I(x,y)\vert dxdy} \\ \sigma_{y}^{(I)}=\frac{\iint_{\Omega} (y-\bar{y})^{2}\vert I(x,y)\vert dxdy}{\iint_{\Omega} \vert I(x,y)\vert dxdy} \\ BQSV^{(I)}=\sigma_{x}^{(I)}+\sigma_{y}^{(I)} \end{matrix}$ | (9) |
| --- | --- | --- |

Here, *I(x, y)* denote the two-dimensional intensity distribution of the optical beam in the transverse plane, and Ω represent the spatial domain corresponding to the effective beam region. The intensity-weighted centroid *(x̄, ȳ)* is calculated from the optical field image *I(x, y)* of the propagating vortex beam, recorded with a spatial resolution of 3480×2160 pixels at a fixed axial position *Z* . $\sigma$*_x_* and $\sigma$*_y_* quantifies the energy-weighted lateral spread of the beam along the x and y direction, respectively, enabling analysis of asymmetric or non-circular beam distributions. The total BQSV provides a scalar descriptor of the overall transverse extent and size of the beam by combining the $\sigma$ along two orthogonal directions.

Based on the mechanism, we systematically investigate the influence of changes in the position, intensity, and size of the light spot in the transverse plane on the BQSV. Notably, BQSV incorporates both the geometric offset of pixels from the centroid and their corresponding intensity weights, making it a second-order statistic primarily governed by the geometric scale of the light spot. For a fixed light spot size, a global translation of the spot within the imaging plane does not alter the relative pixel coordinates with respect to the intensity-weighted centroid. Consequently, the spatial offset terms (*x−*$\bar{x}$)^2^ and (*y−*$\bar{y}$)^2^ remain invariant during the weighted summation. Similarly, under uniform scaling of the overall brightness of the light spot, although the pixel intensities change, the intensity terms appear simultaneously in both the numerator and denominator of the normalized weighting process and cancel each other out, rendering the resulting BQSV independent of the absolute intensity. This characteristic is verified in Figure S7, where light spots with identical lateral sizes exhibit highly consistent BQSV values despite variations in spatial position and intensity.

**Figure S7.** Influence of BQSV on the centroid position, and intensity distribution of the light spot. a) Ten beams with the same light spot pixel but different positions or intensities. b) No impact of light spot pixel position or intensity on the orthogonal and overall BQSV values.

Conversely, variations in the lateral size of the light spot lead to a systematic reconfiguration of pixels distribution relative to the centroid. As shown in Figure S8, with increasing light spot size, pixels in the outer region exhibit a markedly larger geometric offset from the centroid. Owing to their large spatial offsets combined with non-zero intensity, these pixels dominate the spatial weighting process. Therefore, variations in BQSV with spot size are predominantly governed by the geometric evolution of the outer region of the spot.

**Figure S8.** Influence of BQSV on the lateral size of the light spot. a) Beams with different light spot pixel. b) Intensity distribution of five beams of different radius along the x-direction of the spot center. c) Impact of light spot pixel lateral size on the orthogonal and overall BQSV values.

**Section S9: Numerical simulations for MHWFs and corresponding BQSV curves**

The far-field distribution is calculated using the commercial software Lumerical FDTD Solutions, with perfectly matched layers applied along the *x, y,* and *z* directions. To balance accuracy and computational cost, the far-field map was sampled on a 71 × 71 grid, and the electric-field distribution is recorded at 2-µm intervals.

**Figure S9**. MHWFs of various liquids and corresponding overall BQSV curves from numerical simulations.

**Section S10: The finite linear combination of power function characterizes the BQSV of MHWF**

For high-density optical field sampling, the response curve exhibits a smooth, single-extreme nonlinear profile with significant asymmetry, which linear or simple quadratic models cannot adequately capture. To better fit this trend and ensure a good distribution of data across the two sides of the fitted model, we employ a finite linear combination of power functions containing integer and fractional exponents to flexibly describe multi-scale nonlinear behaviour while maintaining analytical tractability, as follows:

|  | $f(x)=a_{0}+\sum_{i=1}^{4} a_{i}z^{\alpha_{i}}\text{ },\text{ }\alpha_{i}\in\left\{\frac{1}{2},1,2,3\right\}$ | (10) |
| --- | --- | --- |

where *a_0_* is the constant term, *a_i_* is the fitting coefficient, and *α_i_* is the power exponent. Table S2 summarizes the correlation coefficients from power-law fits of the BQSV curves for different liquid medium.

**Table S2**: The fitting coefficient and coefficient of determination

| **Medium** | *a_o_* | *a_1_* | *a_2_* | *a_3_* | *a_4_* | *R^2^* |
| --- | --- | --- | --- | --- | --- | --- |
| **DI water** | 90465.34399 | -17677.20573 | 1015.21182 | -1.27726 | 0.000971469 | 0.99801 |
| **Ethanol** | 50077.671131 | -9420.37242 | 525.34021 | -0.63694 | 0.000481006 | 0.99227 |
| **IPA** | 40292.80130 | -7355.14643 | 399.43076 | -0.46480 | 0.00034364 | 0.99269 |
| **DMSO** | 148614.71776 | -26764.68952 | 1417.25829 | -1.52044 | 0.000991981 | 0.99238 |

As shown in Figure S10a-d, the model derived from the MHWF axial evolution accurately captures both the global curvature of the data and the subtle local deviations, demonstrating consistent agreement across the entire fitting range. Furthermore, the detailed residual analysis (Figure S10e-h) shows that the residuals are symmetrically distributed about zero with a mean close to zero and an approximately normal distribution, indicating that the model effectively captures the dominant physical trends. Taking into account the overall fitting performance and the statistical behavior of the residuals, the model demonstrates not only excellent mathematical accuracy but also physical consistency, as it effectively captures the axial optical field evolution of the MHWF, providing a reliable foundation for optical-parameter inversion and physical interpretation based on MHWF.

**Figure S10.** Fitting curves(a-b) and residual analysis(d-f) for different liquid media.

**Section S11: Evolution of the transverse and longitudinal components of OAM along the z-axis**

We adopt the canonical spin-orbital decomposition^[7, 8]^ to investigate the evolution of the OAM contributions, we consider the propagation of a focused vortex beam in IPA as a representative case, as the other investigated liquids, including DI water, ethanol, and DMSO, exhibit analogous focusing and propagation behavior under the same metasurface configuration, with the differences primarily manifested as medium-dependent focal shifts and attenuation. We used Lumerical FDTD software to calculate the full-vector complex electric field **E** (*x, y, z*) and magnetic field **H** (*x, y, z*) from a three-dimensional finite-difference time-domain simulation. The fields were sampled on transverse *xy* planes at 21 axial positions within a ±50 μm range around the focal plane along the propagation direction, corresponding to z = 225.5-325.5μm.

The canonical OAM is based on the spatial-rotation generator ^[9]^ **r**×(−*i* ∇), and therefore provides a direct measure of the OAM imparted by the metasurface-induced vortex phase.

At each propagation position, a local coordinate system is defined as follows:

|  | $\mathbf{r}_{c}=[x-x_{c}(z),y-y_{c}(z),0]$ | (11) |
| --- | --- | --- |

Where *x_c_*(*z*) and *y_c_*(*z*) are the $x$- and $y$-coordinates of the electromagnetic-energy centroid on the transverse plane at position $z$. By defining a local coordinate system for each transverse sampling plane, the origin-dependent extrinsic contribution to the transverse OAM can be effectively removed. This treatment ensures that the calculated OAM components primarily represent the intrinsic OAM carried by the focused vortex field, rather than an artificial contribution associated with the choice of the global coordinate origin.

Based on the extracted full-vectorial complex electromagnetic fields, the *i*-th Cartesian component of the canonical OAM density is defined as follows^[9, 10]^ :

|  | $L_{i}^{can}(x,y,z)=\frac{1}{4\omega_{0}}\mathrm{Im}[\varepsilon\sum_{j} E_{j}^{*}(\mathbf{r}_{c}\times\nabla)_{i}E_{j}+\mu\sum_{j} H_{j}^{*}(\mathbf{r}_{c}\times\nabla)_{i}H_{j}]$ | (12) |
| --- | --- | --- |

where *i=x, y, z* represents the two transverse directions, *x* and *y*, and the longitudinal propagation direction, *z*, respectively, while *j=x, y, z* denotes the three Cartesian components of the electric and magnetic fields.

To focus on the evolution of OAM during propagation, we further calculate the OAM components carried per unit electromagnetic energy:

|  | $l_{i}(z)=\frac{\iint\mathrm{Im}\left[\varepsilon\sum_{j} E_{j}^{*}(\mathbf{r}_{c}\times\nabla)_{i}E_{j}+\mu\sum_{j} H_{j}^{*}(\mathbf{r}_{c}\times\nabla)_{i}H_{j}\right]dA}{\iint[\varepsilon\left\vert\mathbf{E}\right\vert^{2}+\mu\left\vert\mathbf{H}\right\vert^{2}]dA}$ | (13) |
| --- | --- | --- |

Correspondingly, the calculated evolution of the transverse and longitudinal OAM components with propagation distance is shown in Figure S11a and b, respectively. Over the entire propagation range of z=222.1-322.1 μm, the two transverse OAM components, *l*_x_ and *l*_y_, remain at the 10^-3^ level, whereas the longitudinal component *l*_z_ consistently stays close to the designed topological charge of *l*=5. The ratios of the two transverse components to the longitudinal component, R*_x_* and R*_y_*, are both below approximately 0.075% (Figure S11c). These results indicate that during propagation in a liquid medium with a complex dielectric response, the OAM carried per unit electromagnetic energy of the focused vortex field is concentrated in the longitudinal component, while the contribution of the transverse OAM relative to the longitudinal component is negligible. In other words, the complex dielectric response of the liquid does not cause a redistribution of the longitudinal-to-transverse OAM, nor does it disrupt the dominant angular momentum direction determined by the vortex phase in the vortex field. The longitudinal OAM carried per unit electromagnetic energy remains almost constant throughout the propagation range and is always close to the designed topological charge of *l*=5, indicating that the intrinsic OAM structure of the focused vortex field is maintained during liquid propagation, and its OAM is stably distributed mainly along the direction of optical field propagation.

Additionally, to further clarify the influence of the liquid extinction coefficient on the OAM, we compared the longitudinal OAM with and without absorption. As shown in Figure S11d, both the electromagnetic energy and the longitudinal OAM decay according to the Beer-Lambert factor exp(-αz), where α=4πk/λ_0_. Therefore, the integrated OAM ratio $L_{z,k}^{can}/L_{z,0}^{can}$ almost overlaps with exp(-αz). In contrast, the ratio $l_{z,k}/l_{z,0}$ remains 1 throughout the propagation range. This result indicates that the extinction coefficient reduces the electromagnetic energy and the longitudinal OAM carried by the optical field, but does not change the longitudinal OAM carried per unit electromagnetic energy.

**Figure S11.** Evolution of the transverse and longitudinal OAM components along the propagation direction. a) Transverse and b) Longitudinal OAM carried per unit electromagnetic energy. c) The ratio of transverse to longitudinal OAM. d) Extinction coefficient on the OAM.

**Section S12: Comparison of focused vortex beam within the MHWF framework** **and focused Gaussian beam**

To elucidate the role of the vortex phase in refractive-index sensing within the MHWF framework, we compare the performance of the focused vortex beam generated by the metasurface designed in this work with that of the focused Gaussian beam generated by a metalens without an imposed vortex phase. The redesigned metalens is shown in Figure S12a and employs the same eight discrete meta-atom structures as those shown in Figure 2b. The metalens has the same operating wavelength (637 nm), diameter (70 μm), and focal length (200 μm) as the focused-vortex metasurface presented in this work. In the simulation, both metasurfaces maintain the same simulation region size, boundary conditions, and mesh size. The calculated electric field intensity distributions of the metalens in the *x-z* and *x-y* planes, is shown in Figure S12b. The metalens forms a typical focused light field at a preset axial position. Furthermore, Figure S12c presents the electric field intensity distribution at representative positions as the focused Gaussian beam generated by the metalens propagates through DI water, ethanol, IPA, and DMSO. Accordingly, we calculate the BQSV values of the focused Gaussian and vortex beams at different axial propagation positions in media, as shown in Figure S12d. At each axial position, the BQSV values of both beams exhibit a strong linear dependence on the refractive index. To quantitatively compare the refractive-index sensitivities of the two beams, we define the sensitivity enhancement factor (*S*_VB/GB_) as the ratio of the refractive-index sensitivity of the vortex beam to that of the Gaussian beam. As shown in Figure S12e, the *S*_VB/GB_ is greater than 1 at each propagation position, indicating that introducing a vortex phase enhances the response of BQSV to changes in the medium's refractive index. The result further demonstrated that the vortex phase increased the discriminability among the axial BQSV trajectories by inducing more pronounced radial expansion and propagation-dependent evolution of the ring-shaped optical field.

**Figure S12.** Comparison of Focused Vortex and Gaussian Beam. a) Diagram of metalens. b) The electric field intensity distributions of the metalens in the x-z and x-y planes. c)Electric-field intensity distributions of the focused Gaussian beam in different liquids. d) The BQSV values of the focused Gaussian and vortex beams at different axial propagation positions. e) The sensitivity enhancement factor SVB/GB at each propagation positions.

**Section S13:** **BQSV at different propagation positions for various medium and goodness of fit**

Figure S13 shows the relationship between refractive index and BQSV at different propagation positions, revealing a strong linear correlation across the entire test range. Furthermore, Table S3 summarizes the goodness-of-fit (R^2^) for the corresponding linear fits, which exceeds 0.99 in all cases, indicating that the linear model consistently and accurately captures the mapping relationship between refractive index and BQSV. This result demonstrates that BQSV exhibits stable and predictable response characteristics to changes in the media's refractive index, validating the feasibility and robustness of refractive index inversion based on BQSV obtained from MHWF.

**Figure S13**.The relationship between refractive index and BQSV at different propagation positions.

**Table S3:** Goodness-of-fit R^2^ for different propagation position

| Position(μm) | 200 | 205 | 210 | 215 | 220 | 225 | 230 |
| --- | --- | --- | --- | --- | --- | --- | --- |
| *R^2^* | 0.99777 | 0.99631 | 0.99787 | 0.99647 | 0.99782 | 0.99847 | 0.99927 |
| Position(μm) | 235 | 240 | 245 | 250 | 255 | 260 | - |
| *R^2^* | 0.99858 | 0.99117 | 0.99011 | 0.99003 | 0.99133 | 0.99001 | - |

**Section S14: Validation of the scalar angular-spectrum representation for MHWF**

We take the propagation of the focused vortex beam in ethanol as a representative example, as DI water, IPA, and DMSO exhibit analogous focusing and propagation behavior under the same metasurface configuration, with the differences primarily manifested as medium-dependent focal shifts and attenuation. We used Lumerical FDTD software to calculate the full-vector complex electric field **E** (*x, y, z*) and magnetic field **H** (*x, y, z*) from a three-dimensional finite-difference time-domain simulation. The fields were sampled on transverse xy planes at 21 axial positions within a ±50 μm range (222.1-322.1μm) around the focal plane along the propagation direction. To examine the influence of the nonparaxial longitudinal electric-field component on angular-momentum coupling, we calculated the energy fraction of the longitudinal electric field as:

|  | $\eta_{z}(z)=\frac{\iint\left\vert E_{z}(x,y,z)\right\vert^{2}dA}{\iint\left\vert E_{x}\right\vert^{2}+\left\vert E_{y}\right\vert^{2}+\left\vert E_{z}\right\vert^{2}dA}$ | (14) |
| --- | --- | --- |

As shown in Figure S14a, *η_z_* remains below approximately 0.42%, indicating that more than 99.58% of the electric-field energy is consistently confined to the transverse electric-field components. This result indicates that the optical field is predominantly transverse and supports the validity of the scalar approximation in the present weakly focused regime. The treatment is also consistent with previous studies in which the longitudinal electric-field component is used to evaluate the validity of scalar optical-field descriptions^[11]^. Based on the above validation, the metasurface-generated focused vortex field can be described using the scalar angular-spectrum representation.

The exact angular-spectrum relation for normalized longitudinal wave-vector component is

|  | $K_{z}/K=\sqrt{1-\left(K_{t}/K\right)^{2}}$ | (15) |
| --- | --- | --- |

Where, $K_{t}=\sqrt{k_{x}^{2}+k_{y}^{2}}$is the transverse wave-vector magnitude. And *K_t_ /K* is defined as the normalized transverse wave-vector magnitude, which represents the angular spread of the angular-spectrum components and serves as the paraxial parameter for the paraxial expansion. Geometrically, it satisfies *K_t_ /K*=sin*θ*, where *θ* is the angle between the wave vector and the optical axis. Applying Taylor expansion, retaining only the second-order terms, we obtain the Fresnel approximation:

|  | $K_{z}/K\approx1-K_{t}^{2}/K^{2}/2$ | (16) |
| --- | --- | --- |

Thus, the angular-spectrum propagation factor becomes

|  | $\exp\left(iK_{z}z\right)\approx\exp(iKz)\exp\left(-i\frac{K_{t}^{2}}{2K}z\right)$ | (17) |
| --- | --- | --- |

which corresponds to the scalar Fresnel diffraction integral in real space:

|  | $U(x,y,z)\approx\frac{e^{iKz}}{i\lambda_{m}z}\iint U_{0}(x^{'},y^{'})\exp\left[i\frac{K}{2z}\left(\left(x-x^{'}\right)^{2}+\left(y-y^{'}\right)^{2}\right)\right]dx^{'}dy^{'}$ | (18) |
| --- | --- | --- |

This expression is equivalent to Eq. 2 in the manuscript, with the complex refractive index embedded in the propagation kernel. To quantitatively evaluate the validity of this approximation, we compare the exact scalar angular-spectrum relation with the Fresnel paraxial expansion as a function of *K*_t_ */K.* As shown in Figure S14b, the two expressions exhibit excellent agreement when *K*_t_ */K* is small, while their difference gradually increases as *K_t_* /*K* becomes larger. Within the investigated refractive-index range, the deviation between the scalar angular-spectrum relation and the Fresnel paraxial expansion remains on the order of 10^−5^ (Figure S14c) confirming that the Fresnel approximation is valid for the present weakly focused propagation regime.

We then further compare the extinction coefficients retrieved using the Fresnel diffraction approximation and the exact scalar angular-spectrum. As shown in Figure S14d, the relative deviation between the extinction coefficients obtained using the two methods remains below 0.07%, confirming that the Fresnel approximation introduces only a negligible deviation in the extinction-coefficient retrieval. This result further indicates that the metasurface designed in this work can be regarded as a microscale planar phase-modulation element with a small geometrical convergence angle, operating in a weakly focused paraxial regime. Therefore, the Fresnel approximation can effectively describe the light-field propagation for MHWF and can be used for quantitative extinction-coefficient retrieval. In addition to maintaining retrieval accuracy, the Fresnel diffraction approximation also offers clear advantages in computational efficiency. The exact scalar angular-spectrum method requires the construction of a full two-dimensional propagation operator at each propagation distance. In contrast, the Fresnel diffraction approximation can be implemented using separable quadratic phase factors, thereby avoiding the explicit storage of the full two-dimensional propagation operator. As shown in Figure S14e and f, the Fresnel diffraction approximation greatly reduces memory consumption and computational time compared with the exact scalar angular-spectrum. Therefore, considering both retrieval accuracy and computational efficiency, the Fresnel diffraction approximation is adopted to describe the propagation of the metasurface-generated focused vortex field and to retrieve the extinction coefficient in this work.

**Figure S14.** Validation of the scalar angular-spectrum representation for MHWF. a) Energy fraction of the longitudinal electric-field component. b) *K_t_/K* and c) absolute deviation between the scalar angular-spectrum and the Fresnel paraxial approximation as a function of *K_t_/K.* d) The extinction coefficients , e) memory consumption and f) total computational time using the Fresnel diffraction approximation and the angular-spectrum.

**Section S15: Potential applications of MHWF**

Liquids are ubiquitous substances in both natural and industrial systems, and their optical properties directly reveal their electromagnetic response. The real part of the refractive index controls phase velocity, refraction, and wavefront evolution of light, while the imaginary part describes the energy loss caused by absorption related to molecular vibrations and composition. Both complement each other and together constitute a complete description of the dielectric response of liquids. Therefore, accurate and simultaneous determination of *n* and *k* constitutes a fundamental requirement for quality control, chemical composition analysis, and process monitoring, and underpins critical analytical and decision-making capabilities across a broad range of applications, including water quality analysis, hazardous substance detection, medical diagnostics, and manufacturing and materials evaluation.

**Figure S15**.Potential applications of quantifying liquid complex refractive index parameters n and k.

**References**

1.	Hale GM, Querry MR. *Appl.Opt.* **1973**, 12, 555-563.

2.	Segelstein DJ. University of Missouri--Kansas City **1981**.

3.	Skolunov A. *Fibre.Chem.* **1997**, 29, 367-373.

4.	Lucarini V, Peiponen K-E, Saarinen JJ, Vartiainen EM. Springer **2005**.

5.	Dickey FM. CRC press **2018**.

6.	Siegman AE. Spie **1990**. 1224, 2-14.

7.	Bliokh KY, Bekshaev AY, Nori F. *New Journal of Physics* **2017**, 19, 123014.

8.	Bliokh KY, Nori F. *Physics Reports* **2015**, 592, 1-38.

9.	Bliokh KY, Dressel J, Nori F. *New Journal of Physics* **2014**, 16, 093037.

10.	Bliokh KY, Bekshaev AY, Nori F. *Physical review letters* **2017**, 119, 073901.

11.	Kulce O, Onural L, Ozaktas HM. *Journal of Modern Optics* **2016**, 63, 2382-2391.
